# Supplementary material for: Quantitative Physiology and Proteome Adaptations of Bifidobacterium breve NRBB57 at Near-Zero Growth Rates
Source: Microbiol Spectr. 2023 May 15;11(3):e02568-22. doi: 10.1128/spectrum.02568-22 (PMC10269484; doi:10.1128/spectrum.02568-22)
Supplement: Supplemental file 1 — Supplemental material. Download spectrum.02568-22-s0001.pdf, PDF file, 0.4 MB [file spectrum.02568-22-s0001.pdf]

Title: Quantitative physiology and proteome adaptations of *Bifidobacterium breve* NRBB57 at near-zero growth rates

Authors: Angela Rocio Ortiz Camargo[a], Oscar van Mastrigt[a], Roger S. Bongers[b], Kaouther Ben-Amor[b], Jan Knol[b,c], Tjakko Abee[a], Eddy J. Smid[a].

[a]Food Microbiology, Wageningen University & Research, Wageningen, The Netherlands

[b]Danone Nutricia Research, Utrecht, The Netherlands

[c]Laboratory of Microbiology, Wageningen University & Research, Wageningen, The Netherlands

Corresponding author: Eddy J. Smid, PO Box 17, 6700AA Wageningen, The Netherlands. eddy.smid@wur.nl, +31317482834.

Running title: Physiology of *B. breve* at near-zero growth rates

Keywords: retentostat, proteomics, chemostat, stringent response, metabolism, bifidobacteria.

SUPPLEMENTARY FIGURES AND TABLES

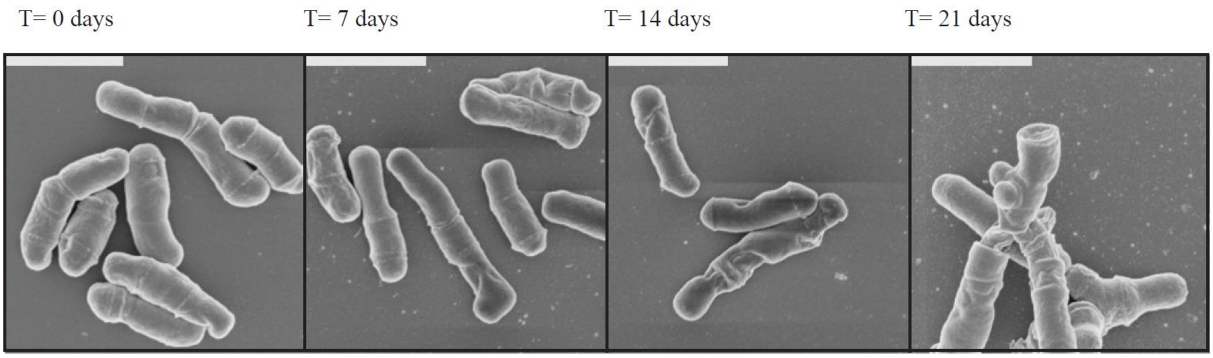

Figure S1: Morphology of *B. breve* NRBB57 cultivated in the retentostat and visualised under scanning electron microscopy. Samples were taken at time 0 which corresponds to the last day of the chemostat at a growth rate of 0.025 h^{-1} and after 7, 14 and 21 days of retentostat cultivation. The length of the white bars in the top left corner of the photographs correspond to 1 µm.

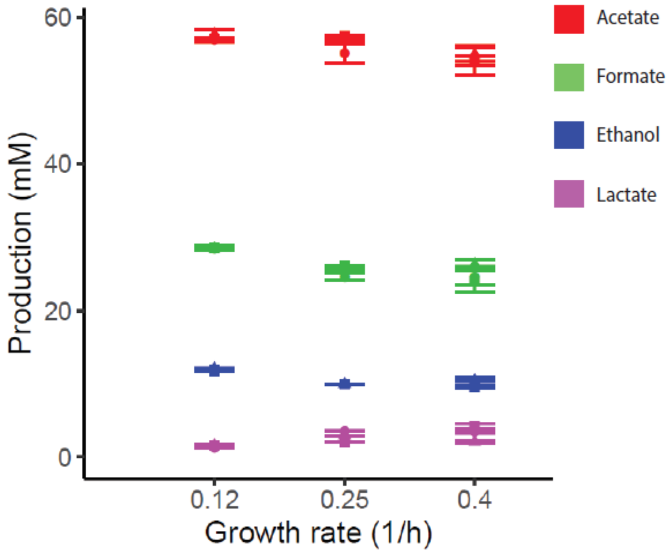

Figure S2: Main products of lactose metabolism at diferrent growth rates in the chemostat over time. Colors represent different metabolites, boxplots show the average of three biological replicates. Results showed a constant concentration of acetate, formate, ethanol and lactate.

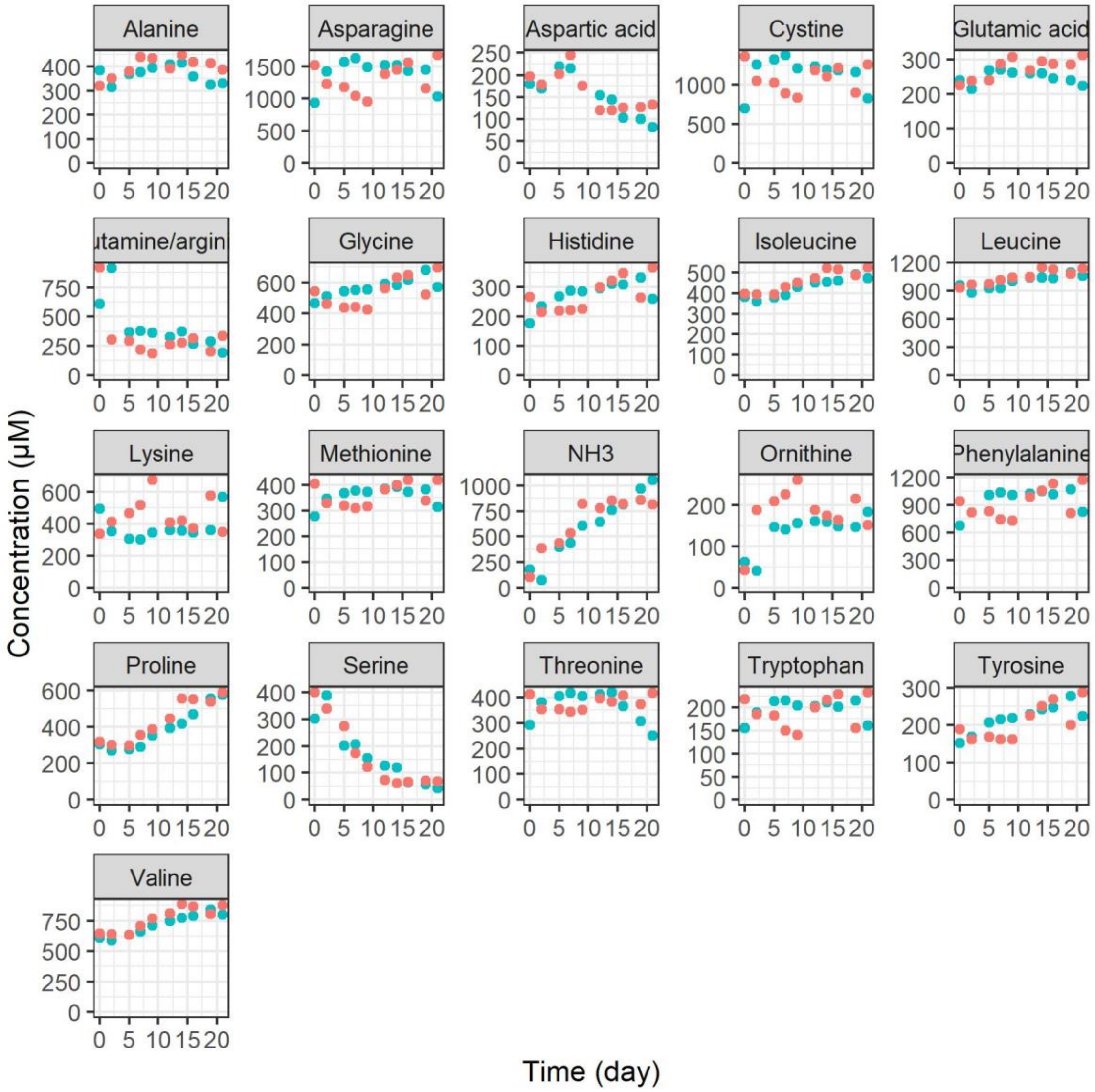

Figure S3: Extracellular amino acids and ammonia concentrations in the retentostat measured with UPLC over time. Colors and symbols represent biological triplicates.The concentrations of these amino acids remained stable throughout the retentostat cultivation.

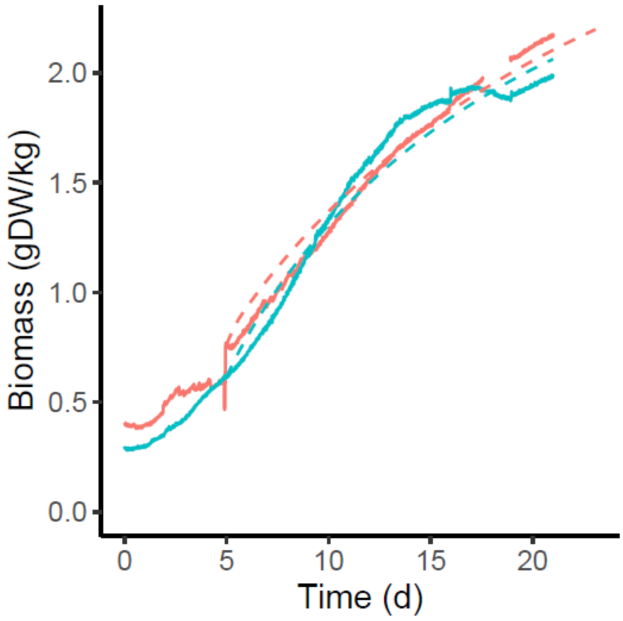

Figure S4: Growth of *B. breve* NRBB57 during the retentostat cultivation. Solid lines represent measured biomass accumulation, dashed lines represent the model predictions of the biomass accumulation. The two colors represent 2 different biological replicates.

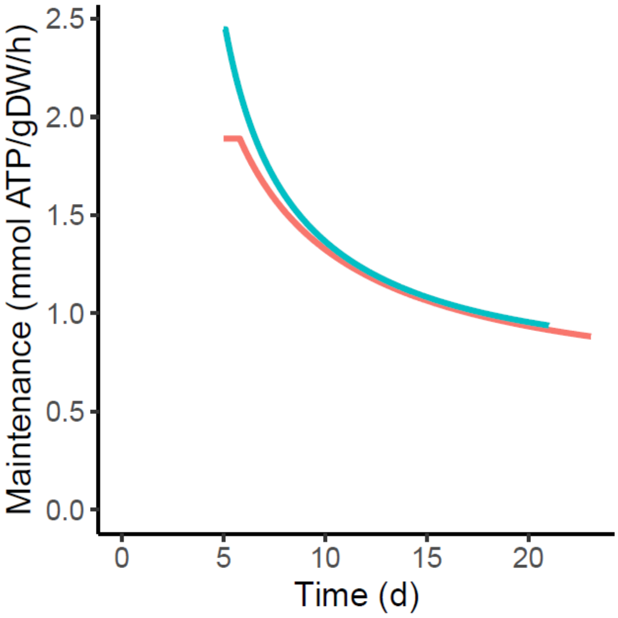

Figure S5: Estimated maintenance coefficient of *B. breve* NRBB57 during the retentostat cultivation, starting at day five. The two colour lines represent 2 different biological duplicates. Mathematical explanation has been explained in the experiment procedures.

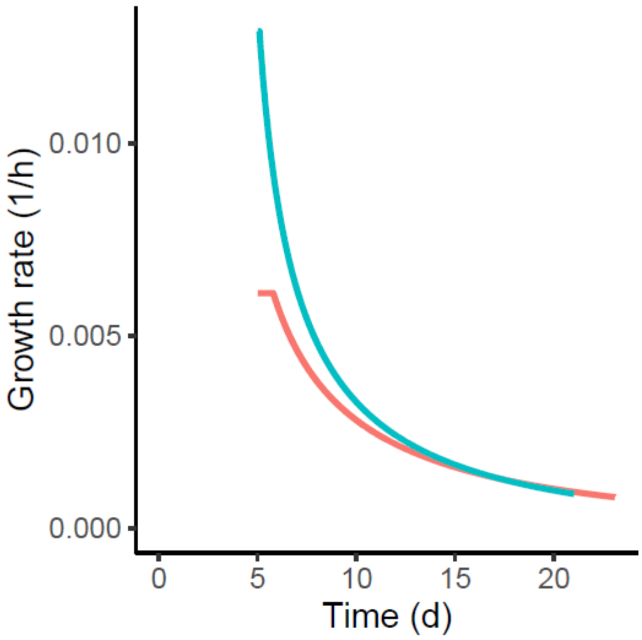

Figure S6: Predicted growth rates starting from day five of the retentostat cultivations.

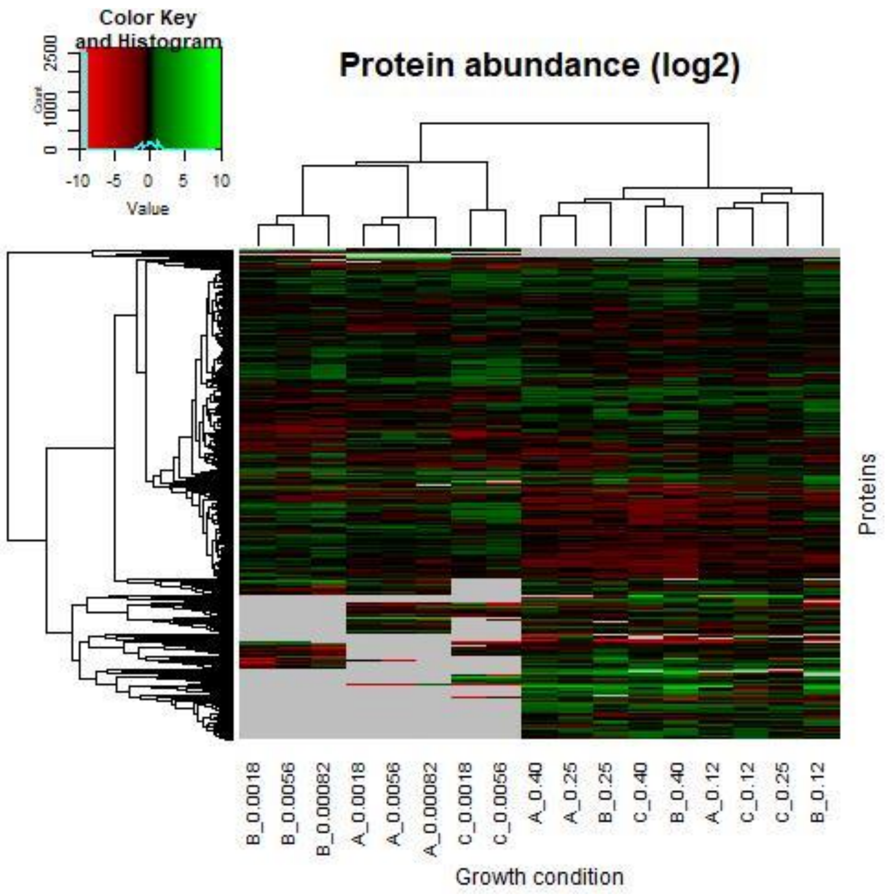

Figure S7: Heat map of the proteome of *B. breve* NRBB57 at different growth rates in chemostat and retentostat cultivation. Values are given as log2 ratio compared to the growth rate of 0.025 h^{-1}. Green cells represent upregulated proteins, red cells represent downregulated proteins. Grey cells represent proteins that could not be quantified. Labels at the growth condition indicate the replicate (A, B, C) and growth rate.

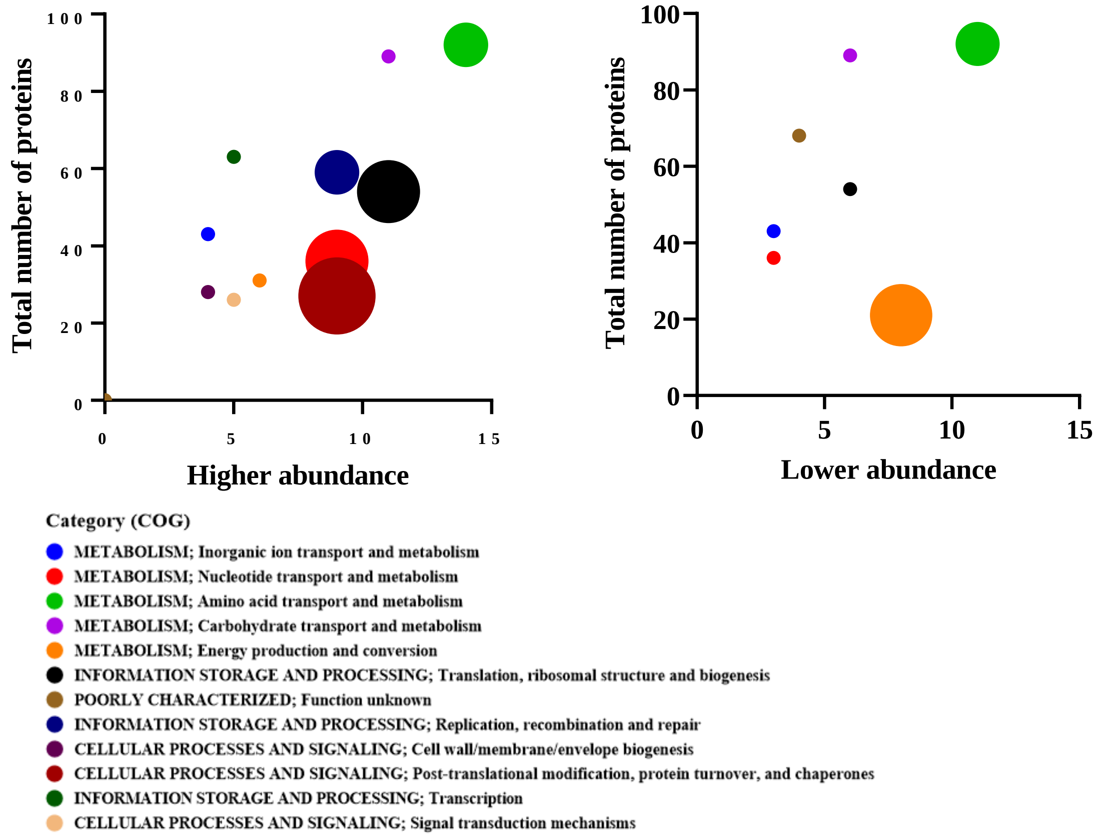

Figure S8: Enrichment of proteins with significant differences in the abundances during the retentostat cultivation. This analysis was carried out with the GSEA-pro v3.0 software. Different colors represent different functional categories. The total number of proteins indicates all the proteins that compose that category versus the proteins that were detected with significant differences. Bubble size represents the score or significance assigned by the software being 3 the biggest size and 0 the smallest size, which indicates that higher scores have more up or down-regulated proteins in relation with the total of proteins in that category.
